# First-principles identification of the charge-shifting mechanism and ferroelectricity in hybrid halide perovskites

**DOI:** 10.1038/s41598-020-76742-7

**Published:** 2020-11-12

**Authors:** Bumseop Kim, Jeongwoo Kim, Noejung Park

**Affiliations:** 1 Department of Physics, Ulsan National Institute of Science and Technology, Ulsan, 689-798 Korea; 2 Department of Physics, Incheon National University, Incheon, 406-772 Korea

**Keywords:** Energy science and technology, Physics

## Abstract

Hybrid halide perovskite solar cells have recently attracted substantial attention, mainly because of their high power conversion efficiency. Among diverse variants, (CH_3_NH_3_)PbI_3_ and HC(NH_2_)_2_PbI_3_ are particularly promising candidates because their bandgap well matches the energy range of visible light. Here, we demonstrate that the large nonlinear photocurrent in *β*-(CH_3_NH_3_)PbI_3_ and *α*-HC(NH_2_)_2_PbI_3_ is mostly determined by the intrinsic electronic band properties near the Fermi level, rooted in the inorganic backbone, whereas the ferroelectric polarization of the hybrid halide perovskite is largely dominated by the ionic contribution of the molecular cation. The spatial charge shift upon excitation is attributed to the charge transfer from iodine to lead atoms in the backbone, which is independent of the presence of the cationic molecules. Our findings can serve as a guiding principle for the design of future materials for halide-perovskite solar cells with further enhanced photovoltaic performance.

## Introduction

Perovskite solar cells (PSCs) have garnered increasing attention as promising optoelectronic devices because of their spontaneous ferroelectricity and the good match between their bandgap energy and the energy range of the solar spectrum^1–5^. Since the first demonstration of PSCs in 2009^6^, substantial progress has been made: the power conversion efficiencies (PCEs) of PSCs, which are the key property with respect to harvesting solar energy, have dramatically increased from 3.8% to 25.2% in this period^6–10^. Recently, numerous attempts to tailor the energy bandgap to enable harvesting of a wider range of the solar energy spectrum have been reported^9,10^.

**Figure 1:**
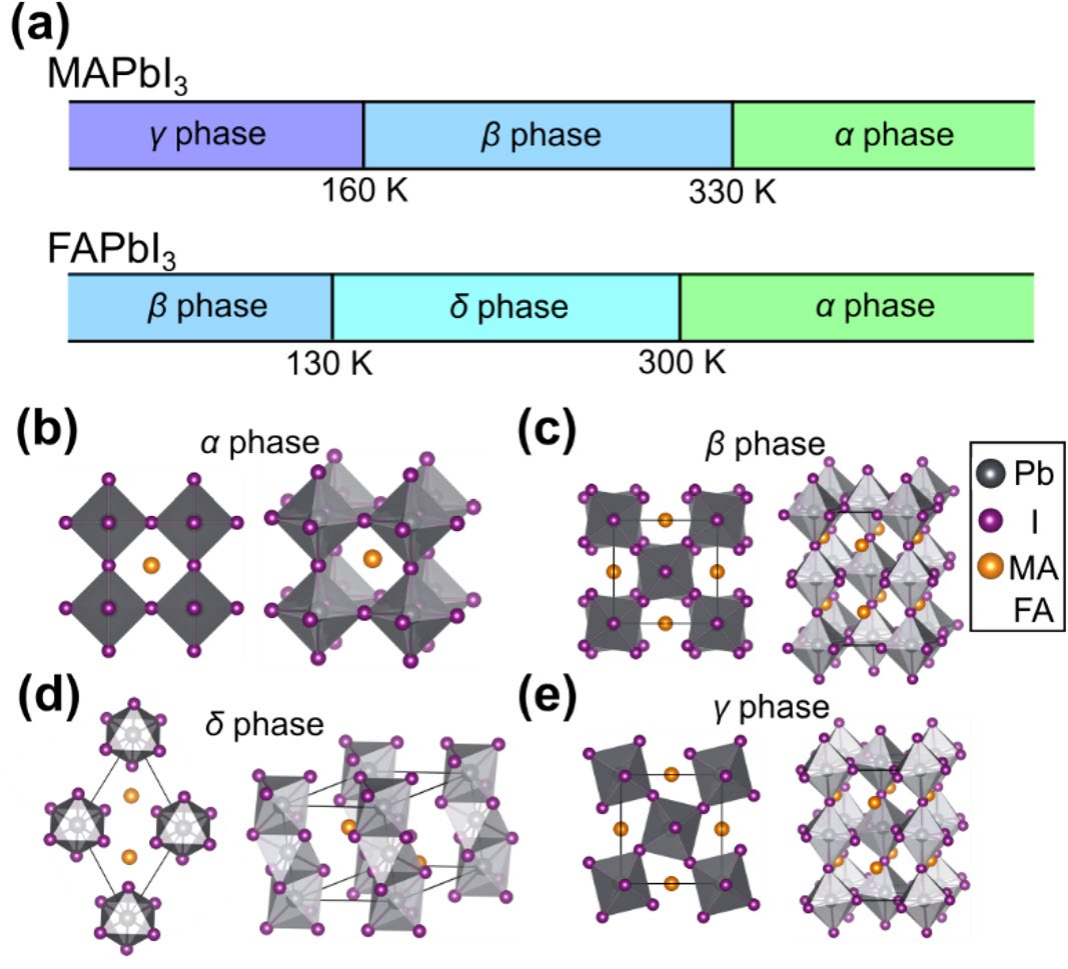
(**a**) The phase variations of MAPbI_3_ and FAPbI_3_ at various temperatures. There are four different phases: (**b**) the cubic alpha (*α*) phase, (**c**) the tetragonal beta (*β*) phase, (**d**) the trigonal delta (δ) phase, and (**e**) the orthorhombic gamma (γ) phase. (**b**)–(**e**) Gray and purple spheres indicate lead and iodine atoms, respectively. The methylammonium (MA) or formamidinium (FA) molecular units are represented by orange spheres.

Among various materials for PSCs, the halide perovskites, CH_3_NH_3_PbI_3_ (methylammonium lead iodide, MAPbI_3_) and HC(NH_2_)_2_PbI_3_ (formamidinium lead iodide, FAPbI_3_), are the most promising candidates in terms of optical absorption and carrier mobility^1,4^. They undergo successive structural phase transitions as their temperature is varied, as illustrated in Fig. 1a^4,11,12^. In both cases, the alpha phase (*α*), characterized by an ordered cubic structure, is stable at high temperatures (> 330 K) (Fig. 1b). Near or below room temperature (< 300 K), MAPbI_3_ exhibits the beta phase (*β*) with space group *I*4/*mcm* (Fig. 1c), whereas FAPbI_3_ preferentially forms the trigonal delta phase (*δ*) (Fig. 1d). *β*-MAPbI_3_ exhibits spontaneous electric polarization caused mainly by the alignment of the MA molecules along the *z*-axis along with marginal atomic distortion of the Pb–I octahedra. Although the presence of the ferroelectricity has been theoretically predicted in halide perovskites^3,13^, its experimental manifestation is not apparent depending on the experimental details^4,14,15^, and it has not been accurately revealed until recently even using state-of-the-art methods such as second harmonic generation and piezoresponse force microscopy^16–20^. The trigonal *δ*-FAPbI_3_ is composed of one-dimensional needle-like structures and exhibits a large bandgap (2.43 eV), leading to marginal photoactivity^21^. At low temperatures (< 130 K), whereas MAPbI_3_ exhibits an orthorhombic gamma (*γ*) phase consisting of rotated octahedra (Fig. 1e), the beta phase becomes stable for FAPbI_3_. *β*-MAPbI_3_ has been explored as a promising candidate for harvesting solar energy^9^; however, the low photoactivity of δ-FAPbI_3_ has hampered its utilization for the photovoltaic effect. As an alternative, *α*-FAPbI_3_, which possesses a desirable bandgap (1.47 eV), has been demonstrated to exhibit a high PCE when its structural stability at room temperature is enhanced by annealing^21^.

The shift current model, also known as the bulk photovoltaic effect (BPVE), is derived by the intrinsic quantum–mechanical nature of the electronic band structures^22–24^. It is the second-order optical response that has been established through second-order perturbation theory^25^. When electrons are excited from the valence band to the conduction bands in a non-centrosymmetric material, the charge center of the electrons is also shifted, giving rise to a non-linear current. The charge-shifting mechanism is attributed to the difference in the Berry connection between the valence and conduction bands^26^, and real-space mapping of the charge variation under excitation is key to fully elucidating the underlying physics. Although halide perovskites have attracted widespread interest as solar energy harvesters and extensive related research has been performed^3,27–31^, the light–matter interaction mechanism (especially for the shift current mechanism) responsible for the bulk photovoltaic characteristics has not yet been satisfactorily investigated. In particular, a comparison and contrast of electronic properties between MAPbI_3_ and FAPbI_3_, which are the most promising candidates among the known halide perovskites, may concretize the search direction for materials that enhance the light-harvesting efficiency PSCs.

In this letter, we investigate the electronic structure and the optoelectronic properties of *β*-MAPbI_3_ and *α*-FAPbI_3_. In particular, we identify the separate contributions of the Pb–I backbone and molecular-cation effect. As a result of extensive density functional theory (DFT) calculations, we find that, although the ferroelectric polarization sharply depends on the alignment of the molecular cations, the large shift current is mainly determined by the intrinsic band structure of the Pb–I backbone. Specifically, charge-transfer within the backbone from I to Pb atoms produces the large shift current. We also discuss the robust shift current generated irrespective of the variation of the electric polarization in a wide range of frequencies.

## Results and discussion

**Figure 2:**
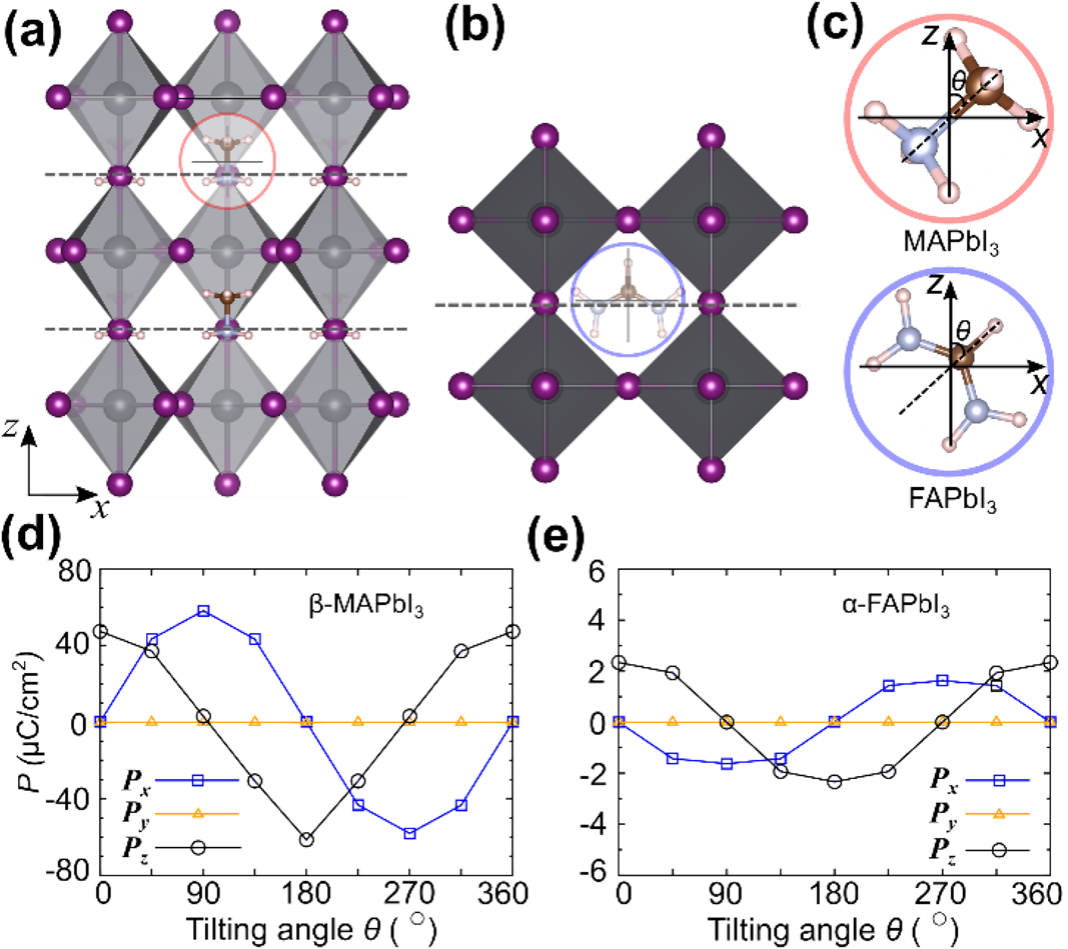
Crystal structures of (**a**) *β-*MAPbI_3_ and (**b**) *α*-FAPbI_3_ with the molecular dipole aligned along the *z*-axis. (**c**) Magnified view of the MA (red circle) and the FA (blue circle) molecular cations in *β-*MAPbI_3_ and *α*-FAPbI_3_ structures, respectively. The tilt of the MA (or FA) molecular cations from the *z*-axis is denoted by *θ*. Electric polarization of (**d**) *β-*MAPbI_3_ and (**e**) *α*-FAPbI_3_ with respect to the tilting angle of the inserted molecules. (**a**)–(**c**) Gray spheres, Pb; purple spheres, I; brown spheres, C; light-blue spheres, N; and pink spheres, H.

We first investigated the microscopic origin of the electric polarization in *β*-MAPbI_3_ and *α*-FAPbI_3_ because ferroelectricity is usually considered a key prerequisite for the BPVE. The detailed crystal structures of both cases are summarized in Tables S1 and S2. As depicted in Fig. 2a,b, these photoactive phases comprise an inorganic frame of octahedral PbI_3_ and organic cations (MA or FA) with the molecular dipole (and also the ferroelectricity) oriented in the *z*-direction^26^. The insets of Fig. 2a,b show that the molecule aligned along the *z*-axis has the *yz* mirror plane. To visualize the effect of molecular orientation, we rotated the molecule in the *xz* plane keeping the inorganic frame fixed and calculated the electric polarization using the Berry phase method^32,33^. The molecular orientation, defined by the tilting angle *θ*, is depicted in Fig. 2c, and the obtained ferroelectricity with respect to the tilting angle is presented in Fig. 2d,e. The electric polarization shows the cyclic behaviors in its *x* and *z* components out of phase by 90°, which implies that the orientation of the molecules is a primary factor influencing the electric polarization in these photovoltaic perovskite materials. As the molecules are rotated in the *xz* plane, the *y*-component of the electric polarization remains intact irrespective of the molecular orientation. As naturally inferred from the close correlation between the molecular orientation and the ferroelectricity, the ionic contribution is dominant in the polarization, whereas the compensating electronic part remains marginal [Fig. S1]. As the orientation of cationic molecule dominantly determines the ferroelectricity of the hybrid halide perovskites, it is questionable whether the pure electronic properties, and related optoelectronic characters (for example, the shift current on excitations), are affected by or independent of the ionic ferroelectricity. Note that the known PCEs of β-MAPbI_3_ and α-FAPbI_3_ are almost comparable^9,10^, despite the large difference in their ferroelectricity. Though extensive studies of the PCEs are necessary to understand the effects of various factors (for example, electron/hole recombination process and grain boundary^1,34,35^) on device characteristics^36,37^, here we can infer that the effect of the ferroelectricity is not essential for the PCEs of these halide perovskite. Later we show that the shift current, the intrinsic electronic nature, is almost independent of the ionic ferroelectricity.

**Figure 3:**
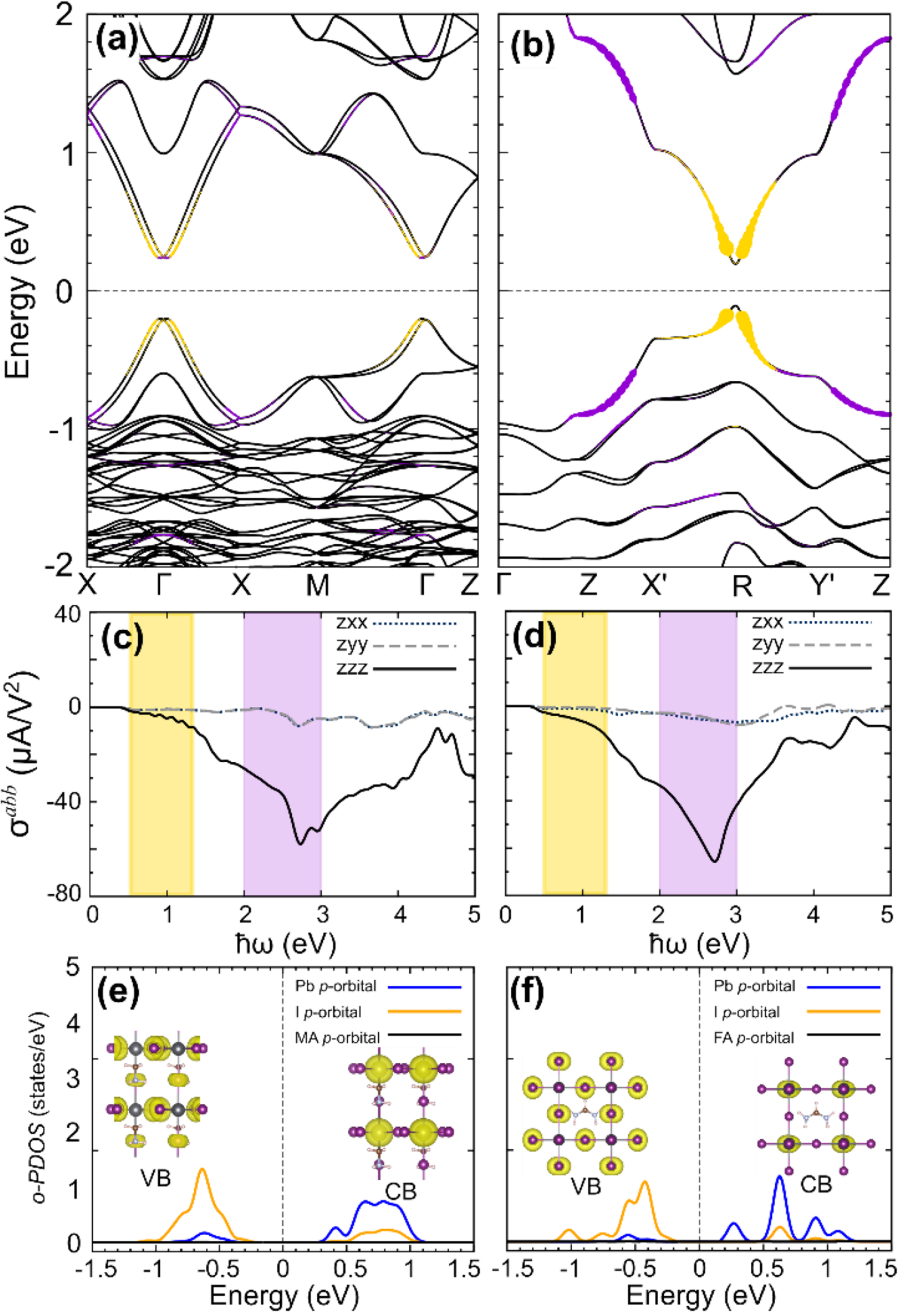
Band structures of (**a**) *β*-MAPbI_3_ and (**b**) *α*-FAPbI_3_ with *z*-directional ferroelectricity. Calculated shift-current spectra ${\sigma }^{abb}$ (*a* and *b* = *x*, *y*, *z*) for (**c**) *β*-MAPbI_3_ and (**d**) *α*-FAPbI_3_ with respect to the frequency of the applied light. Optical active projected density of states (*o-PDOS*) of (**e**) *β*-MAPbI_3_ and (**f**) *α*-FAPbI_3_ corresponding to effective visible-light active range [the yellow dots in (**a**) and (**b**)]. The insets in (**e**) and (**f**) are the real-space representation of the charge density of the initial and the final states. The optical excitations corresponding to the yellow (purple) shift-current range in (**c**) and (**d**) are represented by the yellow (purple) dots in (**a**) and (**b**), respectively. (**a**)–(**b**) The dot size is proportional to the transition rate, and the purple dot size is enlarged five times for clear visualization.

To elucidate the origin of the large BPVE in *β*-MAPbI_3_ and *α*-FAPbI_3_, comparable to the well-known perovskite oxides such as PbTiO_3_ (~ 53 μA/V^2^) and BaTiO_3_ (~ 36 μA/V^2^)^38^, we calculated the electronic structure and optoelectronic properties of the photoactive phases. As illustrated in Fig. 3a, the valence-band maximum (VBM) and the conduction-band minimum (CBM) of *β*-MAPbI_3_ are located near the Γ point in the tetragonal first Brillouin zone [Fig. S2(a)], exhibiting apparent Rashba splittings induced by the *z*-directional electric polarization. For *α*-FAPbI_3_ (Fig. 3b), similar Rashba-split bands appear near the R point [Fig. S2(b)]; however, their splittings are considerably smaller than that of *β*-MAPbI_3_, consistent with the diminutive electric polarization. The effect of Rashba splitting on the density of states is summarized in Fig. S3. Even when the ferroelectricity of *α*-FAPbI_3_ is adjusted by relocation of the molecule, as shown in Fig. S4, the Rashba splittings still remain smaller than those of *β*-MAPbI_3_. Notably, the calculated bandgaps of *β*-MAPbI_3_ (0.46 eV) and *α*-FAPbI_3_ (0.38 eV), which are consistent with the previous theoretical results^39^, are much smaller than the experimental values (1.55 eV for *β*-MAPbI_3_ and 1.47 eV for *α*-FAPbI_3_)^13,40^, which is ascribed to the well-documented bandgap underestimation of standard DFT. We compared the band dispersions and orbital characters obtained by the PBE potential with those obtained by the HSE functional, and the electronic structures shown in Fig. 3 preserve the qualitative validity other than the inherent underestimation of the quasiparticle excitation energy [Fig. S5].

The shift-current spectra of *β*-MAPbI_3_ and *α*-FAPbI_3_, as computed using second-order-perturbation theory, are presented in Fig. 3c,d with respect to the light frequency. Their shift currents increase with increasing photon energy and then reach maximum values (59 μA/V^2^ for *β*-MAPbI_3_ and 67 μA/V^2^ for *α*-FAPbI_3_) at ~ 2.7 eV. Given the tendency of DFT to underestimate bandgaps, we classified the photon energy into two regimes: the effective visible-light active range [0.5–1.3 eV, corresponding to the yellow area in Fig. 3c,d] and the effective ultraviolet (UV) active range [2.0–3.0 eV, corresponding to the purple area in Fig. 3c,d]. The corresponding energy ranges in the band structures are denoted with the same color scheme (Fig. 3a,b). As expected, the effective visible-light-induced shift current is driven by the excitations among band edges, which include substantial Rashba splittings, whereas the effective UV-light contribution is more widely distributed over the whole Brillouin zone. More detailed momentum-resolved shift currents along the high-symmetry lines are presented in Fig. S6 We note that, when the Pb-I backbone is fixed, the energy difference between the ferroelectric and the antiferroelectric states is 21 meV (Fig. S7), indicating the observed ferroelectricity can be reduced in experiment. Nevertheless, the overall trend of the shift current spectrum is well maintained irrespective of the molecular orientation (Figs. S8 and S9).

We now examine the orbital character of the band-edge states responsible for the shift current in the given ranges, for example the effective visible or UV range, as shown in Fig. 3c,d. Here, we define the optical active projected density of states (o-PDOS) by the orbital-projected DOS (PDOS) weighted by the contributions of each band state to the shift current. Detailed description of the definition of the o-PDOS, in comparison with the PDOS, are summarized in supplementary note 1. The optical active PDOS in the effective visible-light active range, which is denoted by yellow dots in Fig. 3a,b, are presented in Fig. 3e,f. The same PDOS in the effective UV-light active range [indicated by the purple dots in Fig. 3a,b] are shown in Fig. S10. Note that the valence (conduction) bands originate mainly from I (Pb) *p*-orbitals, which means the charge shift upon excitation is induced by charge transfer from I to Pb atoms. The orbital character of the initial and final states, as represented in real-space charge densities in the inset of Fig. 3e,f, indeed confirms this character. This result indicates that the role of the cationic molecule (MA or FA) is limited to charge donation and that the large photovoltaic charge shift in these perovskite materials occurs through I–Pb charge transfer within the backbone.

**Figure 4:**
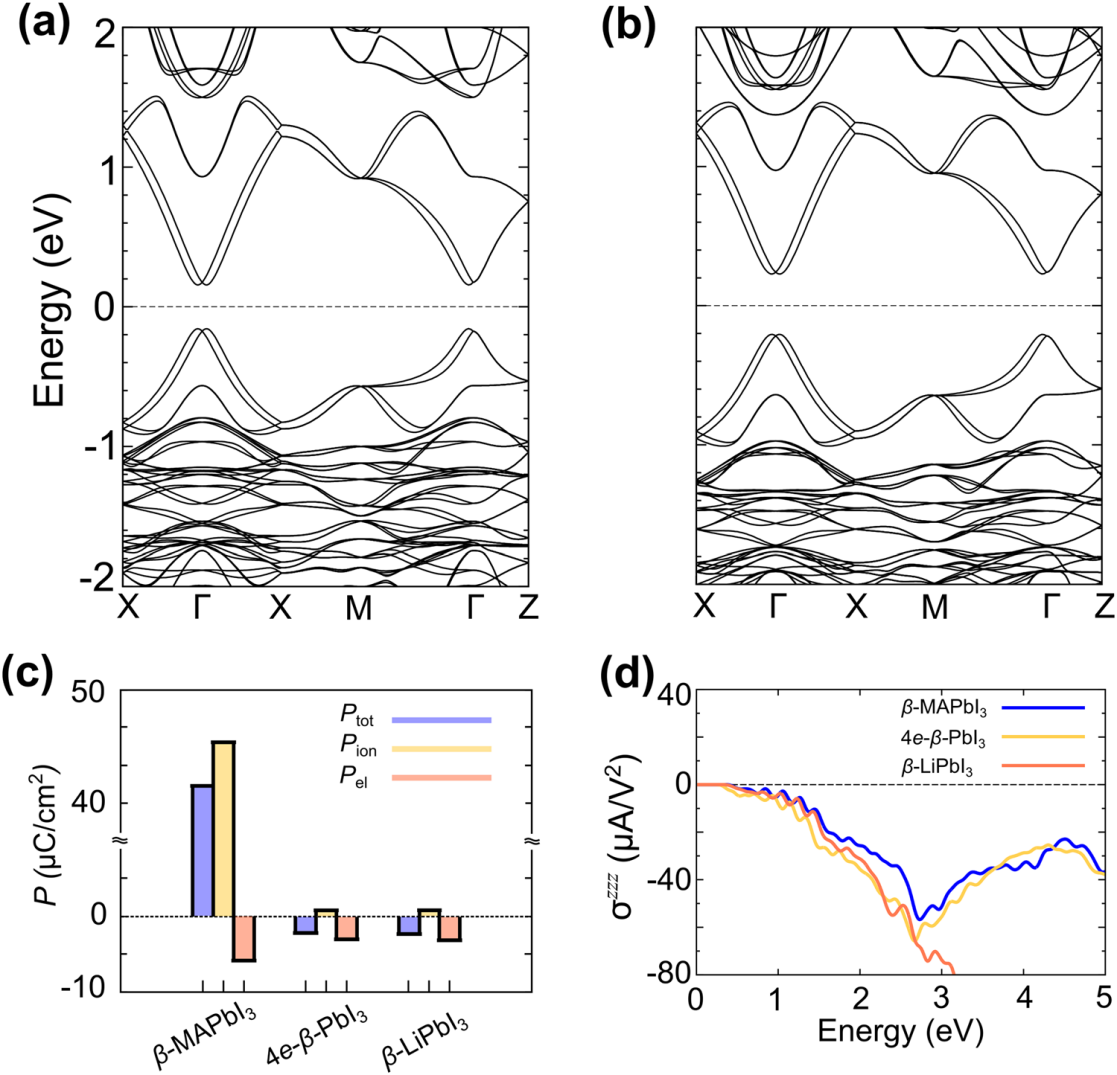
(**a**) Band structures of the beta phase of PbI_3_ (**a**) without a cationic molecule but with the corresponding number of added electrons (4 *e*/cell), referred to as 4*e*-*β-*PbI_3_. (**b**) The same as shown in (**a**) but with Li substituted for the MA molecule, referred to as *β*-LiPbI_3_. (**c**) Electric polarization (*P*_tot_), ionic polarization (*P*_ion_), and electronic polarization (*P*_el_) of *β*-MAPbI_3_, 4*e*-*β-*PbI_3_, and *β*-LiPbI_3_. (**d**) Calculated shift-current spectra for *β*-MAPbI_3_, 4*e*-*β-*PbI_3_, and *β-*LiPbI_3_.

To more explicitly demonstrate that the Pb–I octahedra dominate the generation of the shift current, whereas the MA molecules merely donate electrons to the framework, we calculated the shift current of the four-electron-addition *β*-PbI_3_ (4*e*-*β-*PbI_3_) and *β*-PbI_3_ with Li substituted for MA molecules (*β*-LiPbI_3_). In all of these calculations, the Pb-I frame is kept fixed and Li atoms are substituted in the center of the MA (or FA) molecules. The states near the Fermi level of these two test cases (Fig. 4a,b) are similar to those in the case of *β*-MAPbI_3_ (Fig. 3a). Surprisingly, the spin-split Rashba bands are maintained even in the absence of the MA molecules, which are the main source of the ferroelectricity. This result indicates that the small I-atom displacement along the *z*-axis is mainly responsible for the Rashba splittings in the bands near the Fermi level [Fig. S11]. In the absence of the molecular dipole, the ferroelectric polarization is substantially diminished as shown in Fig. 4c, however, these two test cases produce shift-current spectra similar to the spectrum of *β*-MAPbI_3_ up to the effective UV active range as shown in Fig. 4d. The deviation of the shift current spectrum of *β*-LiPbI_3_ in high energy region is attributed to the presence of the unoccupied Li states above 3 eV. These results clearly imply that the aforementioned shift currents are mainly produced by the inorganic framework (Pb–I octahedra) and that the generated shift current is not proportional to the ferroelectricity. Furthermore, as evidenced by the results for *α*-FAPbI_3_, the Rashba bands are not a prerequisite for a large shift current in halide perovskites. We obtained a similar shift-current spectrum even in the absence of the obvious Rashba splitting in *α*-FAPbI_3_ [Fig. S12].

## Conclusion

In summary, we investigated the electronic band structures and optoelectronic properties of *β*-MAPbI_3_ and *α*-FAPbI_3_ using first-principles methods. We showed that, although the molecular cation is the primary contributor to the ferroelectricity of these halide perovskites, the shift current up to the effective UV active range mostly originates from charge transfer within the Pb–I backbone octahedra. To demonstrate these separate mechanisms, we tested two artificial structures with pure electron doping without a molecular cation and with Li substituted for the cation molecules. We concluded that this ab initio understanding of the sharply distinct roles of the cationic molecules and the backbone inorganic framework can be used as a guiding principle for the future development of related photovoltaic materials.

## Method

Our DFT calculations were performed with the projected augmented plane-wave method^41,42^, as implemented in the Vienna Ab initio Simulation Package (VASP)^43^. The exchange–correlation potential proposed by Perdew, Burke, and Ernzerhof (PBE) was primarily used^44^, however, a nonlocal hybrid functional, the Heyd–Scuseria–Ernzerhof (HSE) functional^45^, was also complementarily used for a cross-check. We used experimentally determined structural parameters for *β*-MAPbI_3_ and *α*-FAPbI_3_ in our calculations. The energy cutoff for the plane-wave-basis expansion was set to 500 eV. We employed 8 × 8 × 6 k-point grids for *β*-MAPbI_3_ and 8 × 8 × 8 k-point grids for *α*-FAPbI_3_ to sample the Brillouin zone. The shift current *J*_*a*_ is a second order response and thus can be expressed in terms of two electric field components and material-dependent response function $({J}_{a}={\sigma }^{abc}{E}_{b}{E}_{c})$. The shift current spectra are expressed by: ${\sigma }^{abc}\left(\omega \right)=\frac{i\pi {e}^{3}}{2{\hslash }^{2}}\int \frac{d\overrightarrow{k}}{8{\pi }^{3}}\sum_{n,m}({r}_{mn}^{b}{r}_{nm;a}^{c}+{r}_{mn}^{c}{r}_{nm;a}^{b})\delta ({\omega }_{mn}-\omega )$ where indices *a*, *b*, and *c* represent Cartesian directions, ${r}_{mn}^{b}$ denotes the velocity matrix elements, and ${r}_{nm;a}^{c}$ denotes the generalized derivatives, which are defined as ${r}_{nm;a}^{c}=\frac{\partial {r}_{nm}^{c}}{\partial {k}_{a}}-i({A}_{mm}^{a}-{A}_{nn}^{a}){r}_{mn}^{c}$ with the Berry connections ${A}_{mm}^{a}$^22,46^. The shift-current spectra were calculated from the maximally localized Wannier function using the WANNIER90 package^47^ with 30 × 30 × 20 k-point grids for *β*-MAPbI_3_ and 50 × 50 × 50 k-point grids for *α*-FAPbI_3_. We show that the Brillouin zone sampling sizes we adopted in our calculation are large enough to give reliable results (Fig. S13).

## Supplementary information

Supplementary Information 1.

## Data Availability

The data that support the findings of this study are available from the corresponding author upon reasonable request.
