# Supplementary material for: First-principles identification of the charge-shifting mechanism and ferroelectricity in hybrid halide perovskites
Source: Sci Rep. 2020 Nov 12;10:19635. doi: 10.1038/s41598-020-76742-7 (PMC7665211; doi:10.1038/s41598-020-76742-7)
Supplement: Supplementary file 1 — Supplementary Information 1. [file 41598_2020_76742_MOESM1_ESM.docx]

***Supplementary information***

**First-principles identification of the charge-shifting mechanism and ferroelectricity in hybrid halide perovskites**

*Bumseop Kim^1^, Jeongwoo Kim^2*^, Noejung Park^1*^*

*^1^Department of Physics, Ulsan National Institute of Science and Technology, Ulsan, 689-798 Korea*

*^2^Department of Physics, Incheon National University, Incheon, 406-772 Korea*

Email: *[noejung@unist.ac.kr](mailto:noejung@unist.ac.kr); [kjwlou@inu.ac.kr](mailto:kjwlou@inu.ac.kr)

**Lattice constants and atomic coordinates of *β*-MAPbI_3_ and *α*-FAPbI_3_**

|  | MAPbI3 | FAPbI3 |
| --- | --- | --- |
| *a* (Å) | 8.849 | 6.3613 |
| *b* (Å) | 8.849 | 6.3613 |
| *c* (Å) | 12.642 | 6.3613 |
| α ( ˚) | 90.0 | 90.0 |
| β ( ˚) | 90.0 | 90.0 |
| γ ( ˚) | 90.0 | 90.0 |

**Supplementary Table 1** Lattice parameters for *β*-MAPbI_3_ and *α*-FAPbI_3_.

| **Atom** | ***β*-MAPbI_3_** | | | ***α*-FAPbI_3_** | | |
| --- | --- | --- | --- | --- | --- | --- |
| **Pb** | 0.0000 | 0.0000 | 0.0000 | 0.0000 | 0.0000 | 0.0000 |
| **Pb** | 0.0000 | 0.0000 | 0.5000 |  |  |  |
| **Pb** | 0.5000 | 0.5000 | 0.5000 |  |  |  |
| **Pb** | 0.5000 | 0.5000 | 0.0000 |  |  |  |
| **I** | 0.0000 | 0.0000 | 0.2472 | 0.5000 | 0.0000 | 0.0000 |
| **I** | 0.0000 | 0.0000 | 0.7472 | 0.0000 | 0.5000 | 0.0004 |
| **I** | 0.5000 | 0.5000 | 0.7472 | 0.0000 | 0.0000 | 0.5000 |
| **I** | 0.5000 | 0.5000 | 0.2472 |  |  |  |
| **I** | 0.2142 | 0.7142 | 0.0046 |  |  |  |
| **I** | 0.7858 | 0.2858 | 0.0046 |  |  |  |
| **I** | 0.2858 | 0.2142 | 0.0046 |  |  |  |
| **I** | 0.7142 | 0.7858 | 0.0046 |  |  |  |
| **I** | 0.2142 | 0.2858 | 0.5046 |  |  |  |
| **I** | 0.7858 | 0.7142 | 0.5046 |  |  |  |
| **I** | 0.2858 | 0.7858 | 0.5046 |  |  |  |
| **I** | 0.7142 | 0.2142 | 0.5046 |  |  |  |
| **C** | 0.5000 | 0.0000 | 0.3520 | 0.5000 | 0.5000 | 0.5695 |
| **C** | 0.0000 | 0.5000 | 0.3520 |  |  |  |
| **C** | 0.5000 | 0.0000 | 0.8520 |  |  |  |
| **C** | 0.0000 | 0.5000 | 0.8520 |  |  |  |
| **N** | 0.5000 | 0.0000 | 0.2420 | 0.6833 | 0.5000 | 0.4752 |
| **N** | 0.0000 | 0.5000 | 0.2420 | 0.3167 | 0.5000 | 0.4752 |
| **N** | 0.5000 | 0.0000 | 0.7420 |  |  |  |
| **N** | 0.0000 | 0.5000 | 0.7420 |  |  |  |
| **H** | 0.5000 | 0.8870 | 0.3650 | 0.5000 | 0.5000 | 0.7412 |
| **H** | 0.5979 | 0.0565 | 0.3650 | 0.8139 | 0.5000 | 0.5676 |
| **H** | 0.4021 | 0.0565 | 0.3650 | 0.7044 | 0.5000 | 0.3155 |
| **H** | 0.0000 | 0.3870 | 0.3650 | 0.2956 | 0.5000 | 0.3155 |
| **H** | 0.0979 | 0.5565 | 0.3650 | 0.1861 | 0.5000 | 0.5676 |
| **H** | 0.9021 | 0.5565 | 0.3650 |  |  |  |
| **H** | 0.5000 | 0.8870 | 0.8650 |  |  |  |
| **H** | 0.5979 | 0.0565 | 0.8650 |  |  |  |
| **H** | 0.4021 | 0.0565 | 0.8650 |  |  |  |
| **H** | 0.0000 | 0.3870 | 0.8650 |  |  |  |
| **H** | 0.0979 | 0.5565 | 0.8650 |  |  |  |
| **H** | 0.9021 | 0.5565 | 0.8650 |  |  |  |
| **H** | 0.5000 | 0.1130 | 0.2290 |  |  |  |
| **H** | 0.5979 | 0.9435 | 0.2290 |  |  |  |
| **H** | 0.4021 | 0.9435 | 0.2290 |  |  |  |
| **H** | 0.0000 | 0.6130 | 0.2290 |  |  |  |
| **H** | 0.0979 | 0.4435 | 0.2290 |  |  |  |
| **H** | 0.9021 | 0.4435 | 0.2290 |  |  |  |
| **H** | 0.5000 | 0.1130 | 0.7290 |  |  |  |
| **H** | 0.5979 | 0.9435 | 0.7290 |  |  |  |
| **H** | 0.4021 | 0.9435 | 0.7290 |  |  |  |
| **H** | 0.0000 | 0.6130 | 0.7290 |  |  |  |
| **H** | 0.0979 | 0.4435 | 0.7290 |  |  |  |
| **H** | 0.9021 | 0.4435 | 0.7290 |  |  |  |

**Supplementary Table 2** Atomic positions for *β*-MAPbI_3_ and *α*-FAPbI_3_

**Supplementary Note 1**

**The definition of optical active projected density of states (o-PDOS)**

We define “optical active PDOS (o-PDOS)” for Fig. 3(e) and (f). To describe an exact definition of the o-PDOS, we need to start with the conventional PDOS and the band-resolved contribution to the shift current.

$\mathrm{PDOS}\left( \varepsilon\right)=\sum_{n} \frac{\Omega_{c}}{8\pi^{3}}\int_{BZ} d\vec{k}\left\langle\psi_{n,\vec{k}} | \hat{P} | \psi_{n,\vec{k}} \right\rangle\cdot\delta(\varepsilon-E_{n}\left( \vec{k} \right))$,

$\sigma_{n}^{abc}\left( \vec{k},\omega\right)=\sum_{m} \frac{i\pi e^{3}}{2\hbar^{2}}\frac{1}{\Omega_{volume}}(r_{mn}^{b}r_{nm;a}^{c}+r_{mn}^{c}r_{nm;a}^{b})\delta(\omega-\frac{E_{n}\left( \vec{k} \right)-E_{m}(\vec{k})}{\hbar})$,

where $E_{n}\left( \vec{k} \right)$ is the energy eigenvalue of the $n$-th band at the Bloch vector $\vec{k}$, $\Omega_{c}$ is the cell volume. The orbital projector can be defined as $\hat{P}=\sum_{Im} \left| \left. \varphi_{Im} \right\rangle\right.\left\langle\left. \varphi_{Im} \right| \right.$ with the target atomic orbitals ($\varphi_{Im}$, the *m*-th orbital of *I*-th atom) to which DOS to be projected. $\sigma_{n}^{abc}$ is the contribution of the $n$-band to the shift current, and the definitions of the shift vectors are provided in the main text. To characterize the contributions of states to the shift current, we devised the following quantity:

o-$\mathrm{PDOS}\left( \varepsilon\right)=\sum_{n} \frac{\Omega_{volume}}{8\pi^{3}}\int_{BZ} d\vec{k}\delta(\varepsilon-E_{n}\left( \vec{k} \right))\cdot\left\langle\psi_{n,\vec{k}} | \hat{P} | \psi_{n,\vec{k}} \right\rangle\cdot\int_{\omega_{1}}^{\omega_{2}} \sigma_{n}^{abc}\left( \vec{k},\omega\right)d\omega$

Figure. 3(e) and (f) are the o-PDOS contributing to the effective visible-light range $(\omega_{1}=0.5 eV, \omega_{2}=1.3 eV)$. The discussions on the energy range corresponding to the visible-light and ultraviolet are provided in the main text. In actual computation, all the delta functions in the equations are represented by Gaussians. For example, $\delta\left( \varepsilon-E_{n}\left( \vec{k} \right) \right)=\frac{1}{\alpha\sqrt{\pi}}e^{-\frac{{(E-E_{n}\left( \vec{k} \right))}^{2}}{2\alpha^{2}}}$

***z*-directional ionic and electronic polarization**

We calculated the ionic and electronic components of the polarization along the *z* direction for *β*-MAPbI_3_ and *α*-FAPbI_3_. As illustrated in Fig. S1(a) and S1(b), the ionic parts prevail over their compensating electronic contribution in both *β*-MAPbI_3_ and *α*-FAPbI_3_. The ionic part of the polarization is more sensitive to the variation of the tilting angle than the electronic part.

**
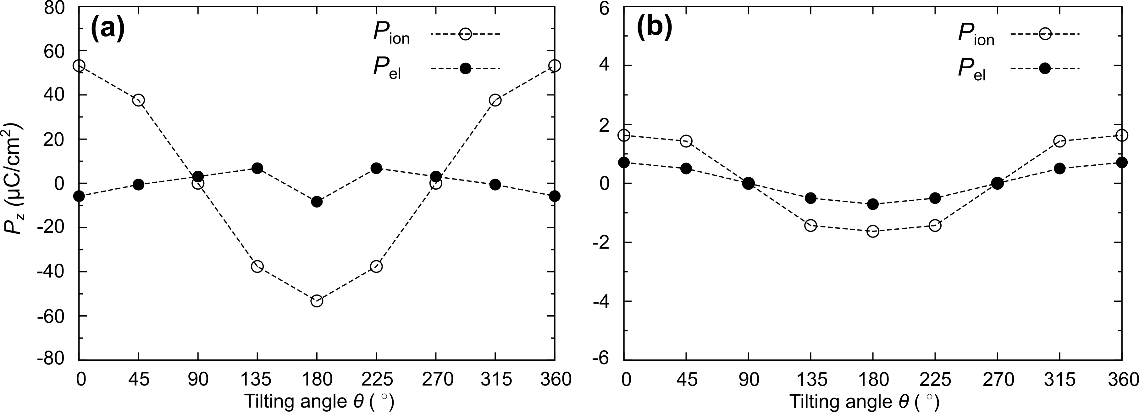
**

**Figure S1** Ionic and electronic components of the electric polarization of (a) *β*-MAPbI_3_ and (b) *α*-FAPbI_3_ in the *z*-direction. This figure is generated by inkscape 0.92.3 version (<https://inkscape.org/ko/release/0.92.3/windows/>) and gnuplot 5.2.7 version (<http://www.gnuplot.info/>)

**Brillouin zone of *β*-MAPbI_3_ and *α*-FAPbI_3_**

The reciprocal vectors for *β*-MAPbI_3_ are $b_{1}=0.1137Å^{-1} \hat{x}$, $b_{2}=0.1137Å^{-1} \hat{y}$ $b_{3}=0.0791Å^{-1} \hat{z}$, as illustrated in Fig. S2(a). The reciprocal vectors for *α*-FAPbI_3_ are $b_{1}=0.1572Å^{-1} \hat{x}$, $b_{2}=0.1572Å^{-1} \hat{y}$, $b_{3}=0.1572Å^{-1} \hat{z}$, as illustrated in Fig. S2(b).


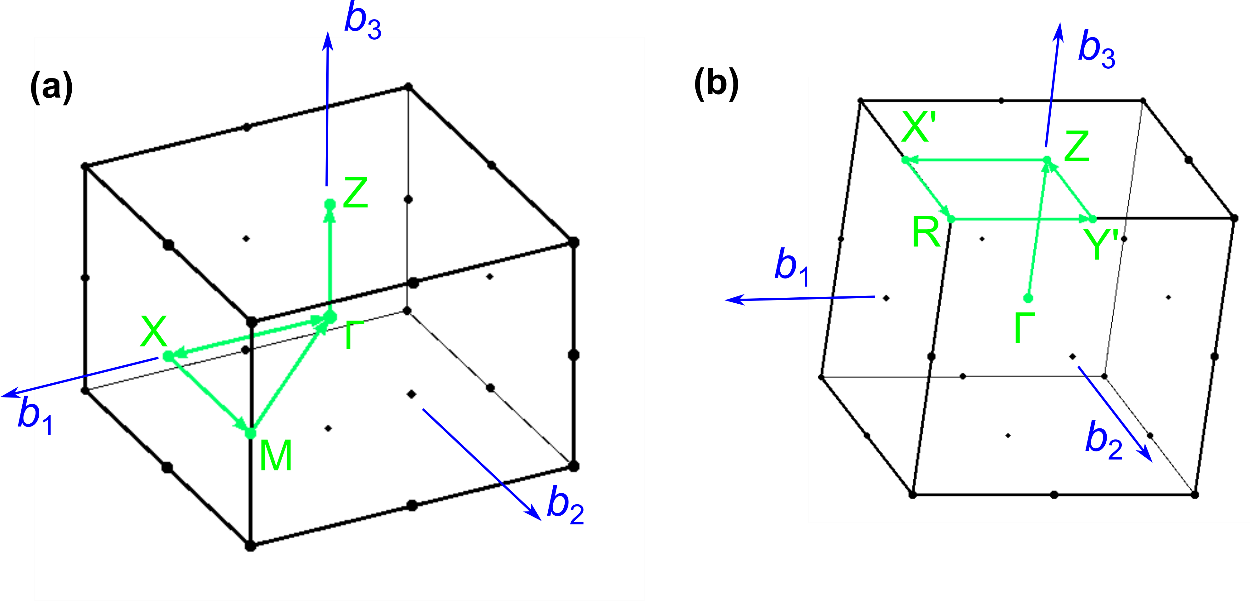


**Figure S2** Three-dimensional bulk Brillouin zone with high-symmetry points indicated in (a) *β*-MAPbI_3_ and (b) *α*-FAPbI_3_. This figure is generated by inkscape 0.92.3 version (<https://inkscape.org/ko/release/0.92.3/windows/>)

**Effect of the Rashba splitting on the density of states of *β*-MAPbI_3_**

We calculated the density of states (DOS) to investigate the effect of the Rashba splitting on it. We compared the electronic structure with and without the Rashba splitting by using the centrosymmetric and inversion-broken configurations, as shown in Fig. S11(d). The Rashba splitting in these cases are very marginal, and conceivable change between two lines cannot be found from the DOSs.

**
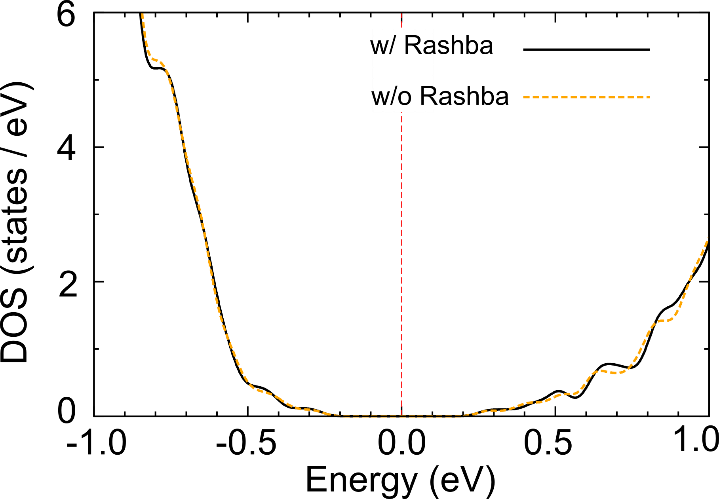
**

**Figure S3** The effect of the Rashba splitting on the density of states (DOS). Black solid (orange dotted) line represent the case with (without) the Rashba splitting band. This figure is generated by inkscape 0.92.3 version (<https://inkscape.org/ko/release/0.92.3/windows/>) and gnuplot 5.2.7 version (<http://www.gnuplot.info/>)

**Variation of the Rashba splitting in *α*-FAPbI_3_**

We investigated changes in the electronic states of *α*-FAPbI_3_ when the cationic molecule is shifted in the *z*-direction, as illustrated in Fig. S4(a) and S4(b). The displacement of the molecule increases the ferroelectricity, leading to the larger Rashba splitting, as denoted by red circles in the R-Y’ lines [Fig. S4(c) and S4(d)]

**
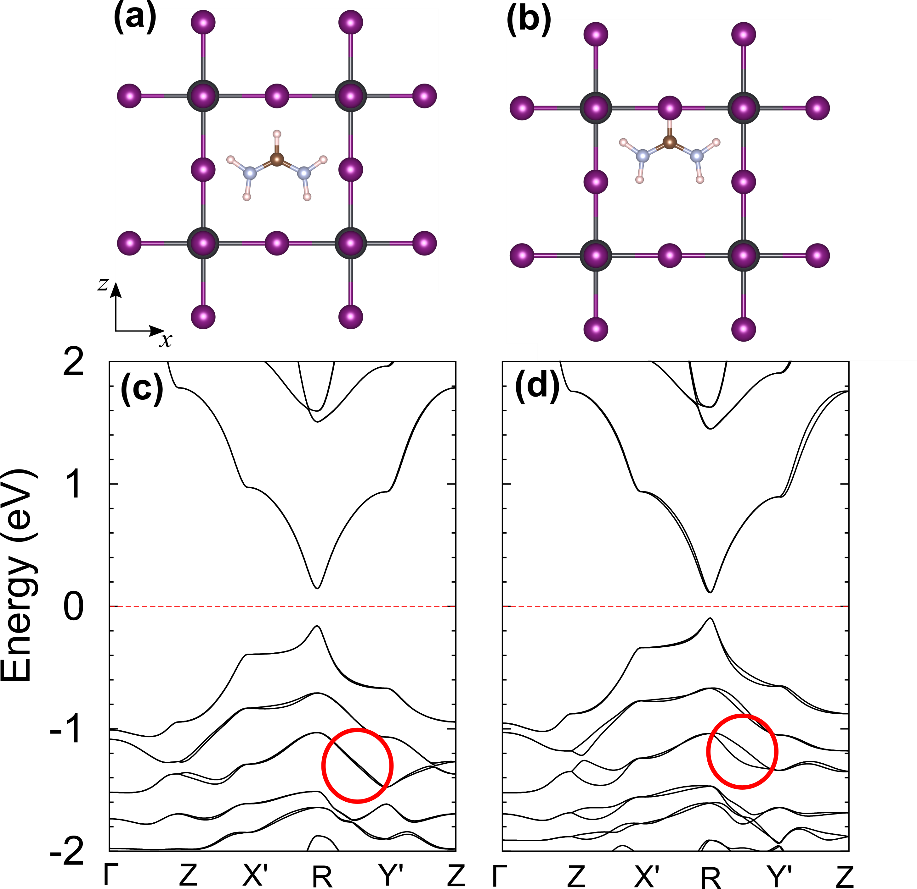
**

**Figure S4** The unit-cell geometry and the atomic structure of (a) *α*-FAPbI_3_ and (b) *α*-FAPbI_3_ with artificial molecular shifting in *z*-direction. Electronic band structures of (c) *α*-FAPbI_3_ and (d) *α*-FAPbI_3_ with artificial molecular shifting in *z*-direction. (c)-(d) red circle indicates the difference in the Rashba splitting of both *α*-FAPbI_3_ and *α*-FAPbI_3_ with artificial molecular shifting. This figure is generated by inkscape 0.92.3 version (<https://inkscape.org/ko/release/0.92.3/windows/>), gnuplot 5.2.7 version (<http://www.gnuplot.info/>), and Visualization for Electronic and Structural Analysis (VESTA) 3.4.5 version (<https://jp-minerals.org/vesta/en/download.html>)

**HSE functional calculation**

We calculated the ground electronic structure and the optical property with the HSE functional for *β*-MAPbI_3_ and *α*-FAPbI_3_. The HSE functional results are qualitatively similar to the PBE results. The band gap of *β*-MAPbI_3_ [Fig. S5(a)] and *α*-FAPbI_3_ [Fig. S5(b)] obtained from the HSE functional is 0.84 and 0.71 eV. The shift current spectra calculated from the HSE functional show the similar pattern observed in the PBE results except for the rigid shift to the high energy corresponding to the band gap correction.

**
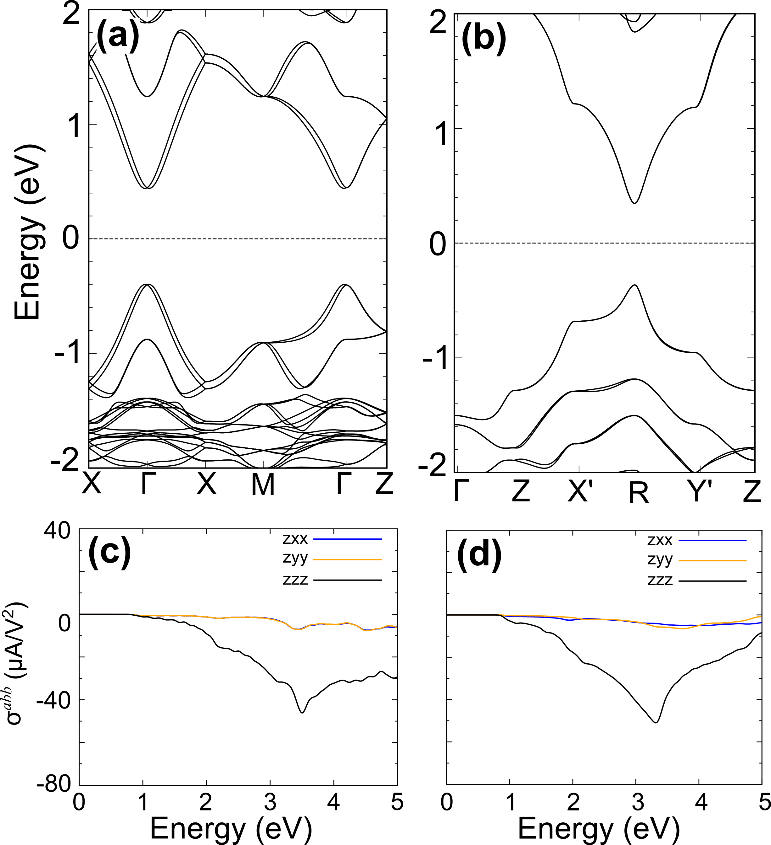
**

**Figure S5** Band structures of (a) *β*-MAPbI_3_ and (b) *α*-FAPbI_3_ with the *z*-directional ferroelectricity. Calculated shift current spectra $\boldsymbol{\sigma}^{\boldsymbol{abb}}$ (*a* and *b*=*x*, *y*, *z*-axis) for (c) *β*-MAPbI_3_ and (d) *α*-FAPbI_3_. (a)-(d) All data are obtained from the HSE functional. This figure is generated by inkscape 0.92.3 version (<https://inkscape.org/ko/release/0.92.3/windows/>) and gnuplot 5.2.7 version (<http://www.gnuplot.info/>)

**Momentum-space-resolved shift current**

We decomposed the shift current spectrum according to the band index and the crystal momentum. We estimated the momentum-resolved shift current

,

where indices *a*, *b*, and *c* represent Cartesian directions, $r_{vc}^{b}$denotes the velocity matrix elements, and $r_{cv;a}^{c}=\frac{\partial r_{cv}^{c}}{\partial k_{a}}-i\left(A_{vv}^{a}-A_{cc}^{a}\right)r_{vc}^{c}$ is the generalized derivatives defined in term of Berry connection $A_{vc}^{a}$. As shown in the Fig. S6(a) and S6(b), the momentum-resolved shift current induced by the effective visible light is localized near the band gap region [yellow line in Fig. S6(a) and S6(b)], while the effective ultraviolet (UV) contribution is widely spread over whole Brillouin zone.

**
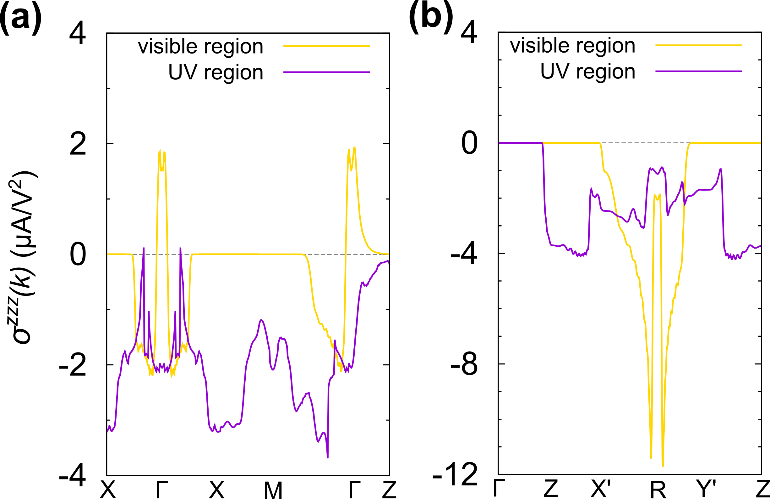
**

**Figure S6** Momentum-resolved shift current spectra along the *z*-axis of (a) *β*-MAPbI_3_ and (b) *α*-FAPbI_3_. This figure is generated by inkscape 0.92.3 version (<https://inkscape.org/ko/release/0.92.3/windows/>) and gnuplot 5.2.7 version (<http://www.gnuplot.info/>)

**Total difference between the ferroelectric and the antiferroelectric states**

We calculated the total energy difference between the ferroelectric and the antiferroelectric configurations for the experimentally determined structure and the fully relaxed structure. As shown in Fig. S8, the energy difference for the experimental structure (exp) is 21 meV, in good agreement with the previous calcaultion^1^. For the fully relaxed structure (relaxed), it increases up to 252 meV because the alternation of the Pb-I frame is accompanied by the MA molecule rotation.

**
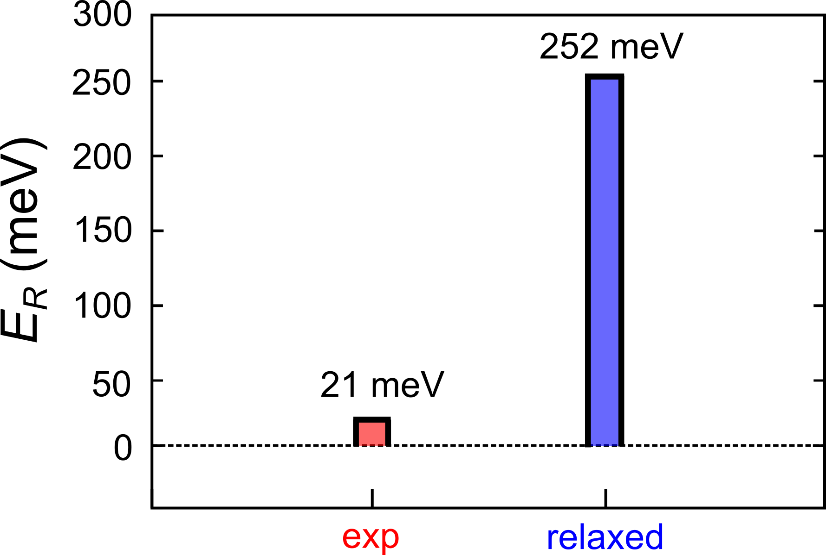
**

**Figure S7** Energy difference between the ferroelectric and the antiferroelectric configurations for the experimentally determined structure (exp) and the fully relaxed structure (relaxed). This figure is generated by inkscape 0.92.3 version (<https://inkscape.org/ko/release/0.92.3/windows/>) and gnuplot 5.2.7 version (<http://www.gnuplot.info/>)

**Electronic structure and shift current upon the cation orientation**

We calculated the electronic structure and the shift current spectra varying the tilting angle *θ* of the cation molecule defined in Fig. 2. The band structures and the shift current spectra of *β*-MAPbI_3_ are quite insensitive to the molecular geometry, because the states near the Fermi level are mostly derived from the Pb-I frame.

**
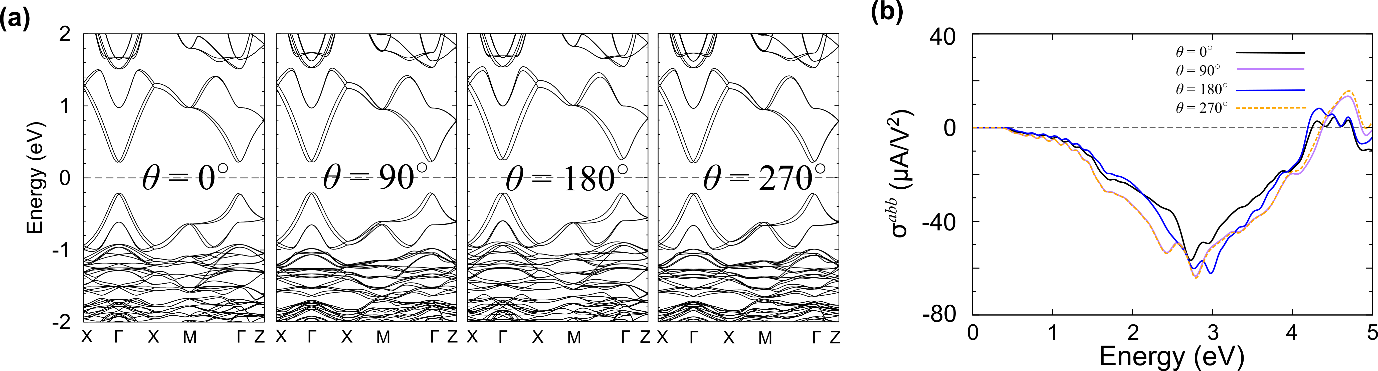
**

**Figure S8** (a) Calculated band structures and (b) shift current spectra $\boldsymbol{\sigma}^{\boldsymbol{zzz}}$ of *β*-MAPbI_3_ with respect to the tilting angle *θ.* This figure is generated by inkscape 0.92.3 version (<https://inkscape.org/ko/release/0.92.3/windows/>) and gnuplot 5.2.7 version (<http://www.gnuplot.info/>)

**band structure and shift current spectra in the relaxed structure**

We calculated the electronic structure and the shift current spectrum of *β*-MAPbI_3_ using the experimental ferroelectric structure (e-ferro), the fully relaxed ferroelectric structure (r-ferro), and the fully relaxed antiferroelectric structure (r-antiferro) as shown in Fig. S9(a). In the relaxed structures, the distortions of the Pb-I frame yield a large change in the electronic band structure as shown in Fig S9(b). These distortions give rise to non-zero $\boldsymbol{\sigma}^{\boldsymbol{zxx}}$ and $\boldsymbol{\sigma}^{\boldsymbol{zyy}}$ components which has not been allowed in the experimental structure as shown in Fig S9(c). Nevertheless, the magnitude of the $\boldsymbol{\sigma}^{\boldsymbol{zzz}}$ component of the relaxed structures is comparable to the one estimated from the experimental structure as illustrated in Fig S9(d).

**
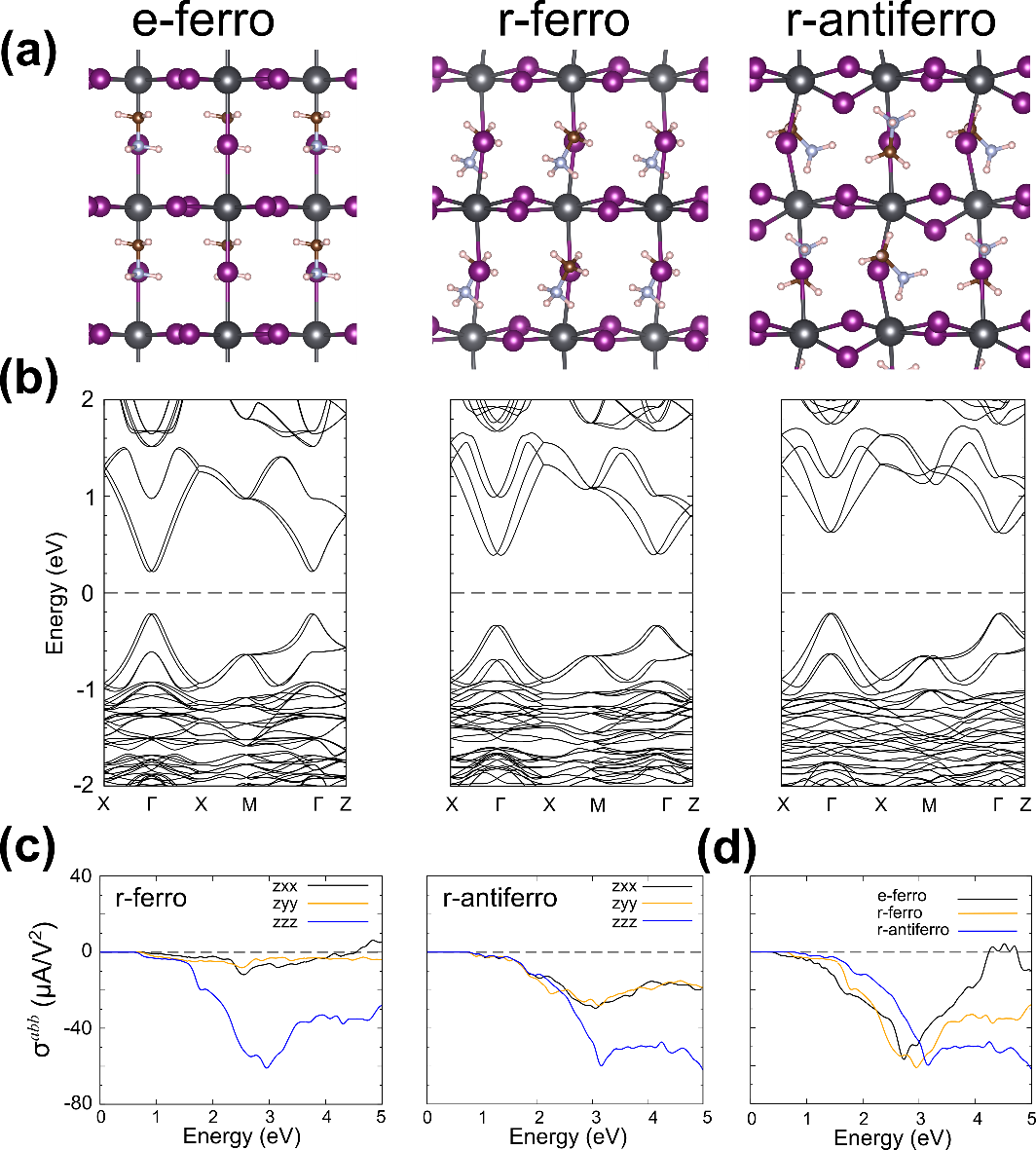
**

**Figure S9** (a) *β*-MAPbI_3_ crystal structures of the experimental ferroelectric structure (e-ferro), the fully relaxed ferroelectric structure (r-ferro), and the fully relaxed antiferroelectric structure (r-antiferro). (b) Band structures of the e-ferro (left), the r-ferro (middle), and r-antiferro (right). (c) Calculated shift-current spectra $\boldsymbol{\sigma}^{\boldsymbol{abb}}$ (*a* and *b* = *x*, *y*, *z*) for the r-ferro and the r-antiferro. (d) Comparison of the z-directional shift current spectra $\boldsymbol{\sigma}^{\boldsymbol{zzz}}$ for the e-ferro, the r-ferro, and the r-antiferro. This figure is generated by inkscape 0.92.3 version (<https://inkscape.org/ko/release/0.92.3/windows/>), gnuplot 5.2.7 version (<http://www.gnuplot.info/>), and Visualization for Electronic and Structural Analysis (VESTA) 3.4.5 version (<https://jp-minerals.org/vesta/en/download.html>)

**Real-space mapping of the effective UV active range shift current**

We investigated the orbital characters corresponding with the effective UV active light which are purple dots in Fig. 3(a) and 3(b) in the main text. As shown in Fig. S10(a), S10(b), and S10(c), the orbital characters of the valence and conduction bands of *β*-MAPbI_3_ are consisting of the iodine and lead’s *p* orbitals, respectively, as same as effective visible light in Fig. 3(e). For *α*-FAPbI_3_, the orbital characters of the valence and conduction bands consist of the iodine and lead’s *p* orbitals, however there are the FA molecule states near 2.7 eV as presented in Fig. S10(d), S10(e), and S10(f).

**
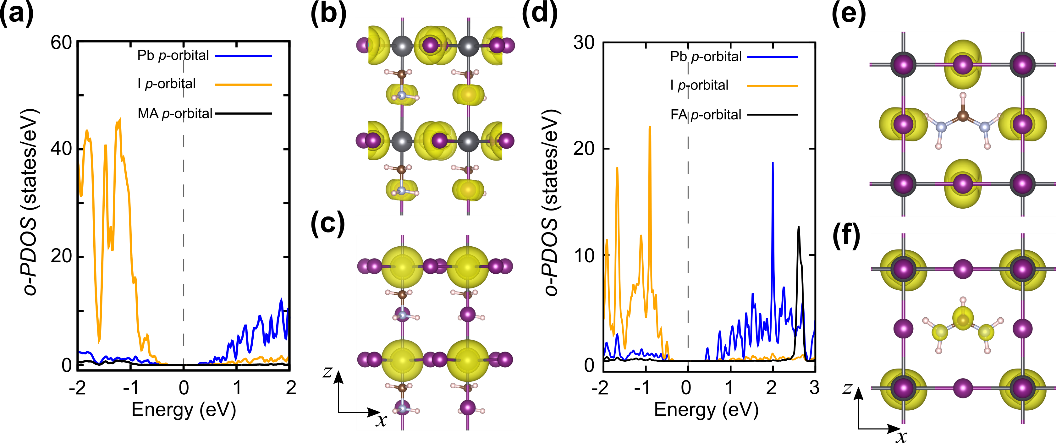
**

**Figure S10** (a) Projected density of states (PDOS) of *β*-MAPbI_3_ corresponding to the effective ultraviolet absorption [the purple regions in Fig. 3(a)] and the real-space representation of (b) the valence and (c) the conduction band charge densities. (d) Projected density of states (PDOS) of *α*-FAPbI_3_ corresponding to the effective ultraviolet absorption [the purple regions in Fig. 3(b)] and the real-space representation of (e) the valence and (f) the conduction band charge densities. This figure is generated by inkscape 0.92.3 version (<https://inkscape.org/ko/release/0.92.3/windows/>), gnuplot 5.2.7 version (<http://www.gnuplot.info/>), and Visualization for Electronic and Structural Analysis (VESTA) 3.4.5 version (<https://jp-minerals.org/vesta/en/download.html>)

**The effect of the atomic distortions of the shift current**

We investigate the effect of the atomic distortion on the shift current spectra of *β*-MAPbI_3_. We tested two different structures: i) the perfect alignment of the spiral Pb-I octahedron [Fig. S11(a)] and ii) the undistorted Pb-I ochaderon [Fig. S11(b)]. As shown in Fig. S11(c) and S11(d), the overall electronic features of *β*-MAPbI_3_ are well maintained in these two testing cases. Nevertheless, the Rashba splitting near the Fermi level almost disappear in the absence of the *z*-axis distortion in Pb-I octahedron [Fig. S11(d)]. The persistent large shift current shows that the production of the photocurrent is rarely affected by the atomic arrangement unless the Pb-I backbone is severely damaged [Fig. S11(e)].

**
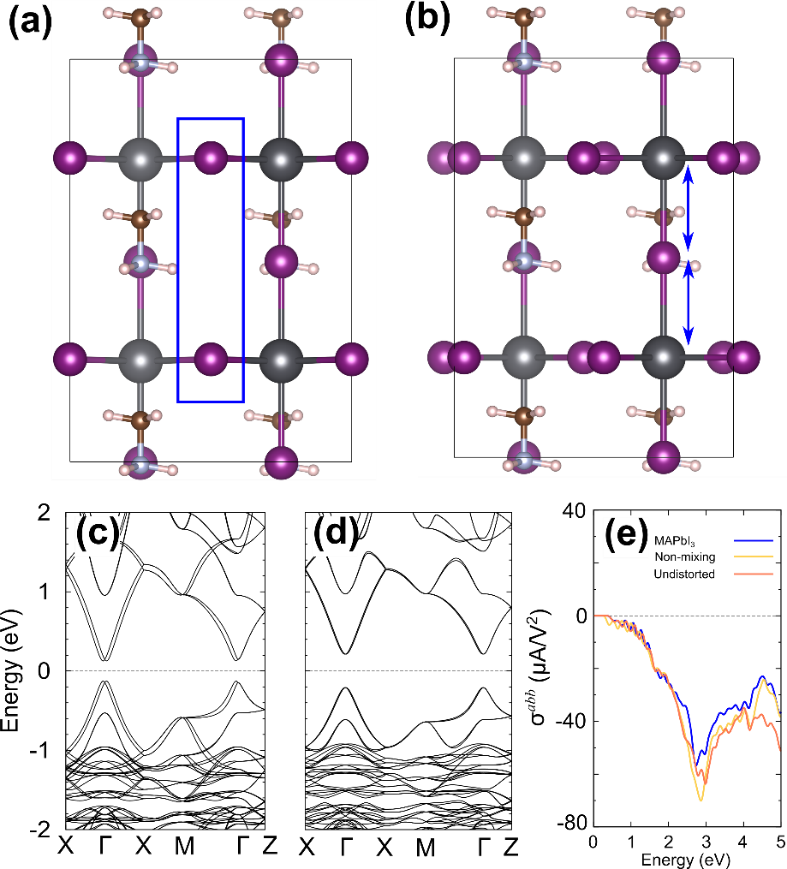
**

**Figure S11** Crystal structures of *β*-MAPbI_3_ (a) with the perfect alignment of the spiral Pb-I octahedron and (b) with the undistorted Pb-I octahedron. Band structures of (c) the perfectly aligned *β*-MAPbI_3_ and (d) the undistorted *β*-MAPbI_3_. (e) Calculated shift current spectra $\boldsymbol{\sigma}^{\boldsymbol{abb}}$ (*a* and *b*=*x*, *y*, *z*-axis) for (c) *β*-MAPbI_3_, the perfectly aligned *β*-MAPbI3, and the undistorted *β*-MAPbI_3_. This figure is generated by inkscape 0.92.3 version (<https://inkscape.org/ko/release/0.92.3/windows/>), gnuplot 5.2.7 version (<http://www.gnuplot.info/>), and Visualization for Electronic and Structural Analysis (VESTA) 3.4.5 version (<https://jp-minerals.org/vesta/en/download.html>)

**Li substitution and electron doping in *α*-FAPbI_3_**

We also investigated the effect of the Li substitution and the electron doping on the shift current in *α*-FAPbI_3_. The electron doing or the Li substitution rarely alters the original electronic structures of *α*-FAPbI_3_ [Fig. S12(a) and S12(b)]. Similar to *β*-MAPbI_3_ of Fig. 4, the absence of the cationic molecules significantly decreases the electric polarization [Fig S12(c)]. Nonetheless, the large shift current spectra are retained up to 3 eV without the molecular dipole [Fig. S12(d)].

**
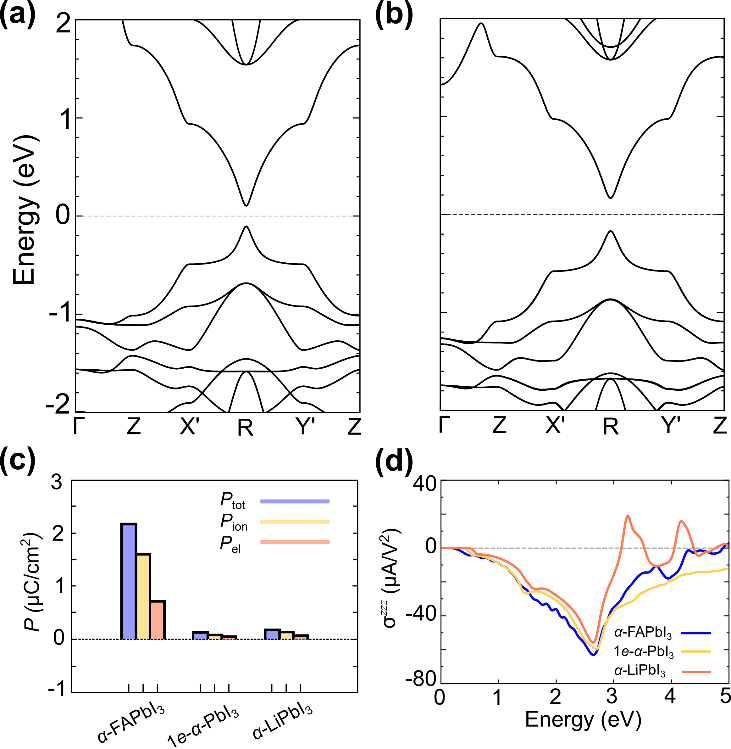
**

Figure S12 Band structures of the alpha phase of PbI_3_ (a) without cationic molecule but with the corresponding number of added electrons (1 *e*/cell), referred to as 1*e*-*α-*PbI_3_. (b) The same as (a) with Li substitution in place of the MA molecule, referred to as *α*-LiPbI_3_. (c) Electric polarization (*P*_tot_), ionic polarization (*P*_ion_), and electronic polarization (*P*_el_) of *α*-FAPbI_3_, 1*e*-*α-*PbI_3_, and *α*-LiPbI_3_. (d) Calculated shift current spectra for *α*-FAPbI3, 1*e*-*α-*PbI_3_, *α*-LiPbI_3_. This figure is generated by inkscape 0.92.3 version (<https://inkscape.org/ko/release/0.92.3/windows/>) and gnuplot 5.2.7 version (<http://www.gnuplot.info/>)

**Variation of the shift current spectra with the k-point sampling size**

We investigated the variation of the shift current spectra with the Brillouin zone sampling size. As shown in Fig. S13(a), the shift current spectrum of the *β*-MAPbI_3_ is successfully estimated by the 15x15x20 **k**-point grid. For the *α*-FAPbI_3_, the 30x30x30 **k**-point grid is large enough to give the correct shift current spectrum as presented in Fig. S13(b). From these results, we can conclude that the sampling sizes we adopted in in Fig. 3(c) and 3(d) are sufficient to investigate the shift current spectra of the *β*-MAPbI_3_ and the *α*-FAPbI_3_.

**
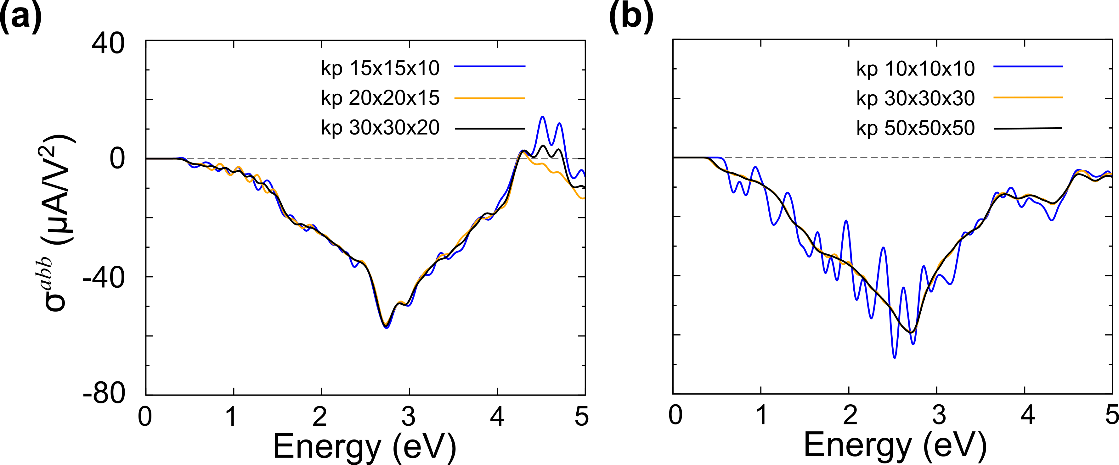
**

Figure S13 Shift current spectra $\boldsymbol{\sigma}^{\boldsymbol{zzz}}$ for the (a) *β*-MAPbI_3_ and (b) *α*-FAPbI_3_ varying the k-point sampling size. This figure is generated by inkscape 0.92.3 version (<https://inkscape.org/ko/release/0.92.3/windows/>) and gnuplot 5.2.7 version (<http://www.gnuplot.info/>)

**References**

1. Zheng, Fan. *et al*. First-Principles Calculation of the Bulk Photovoltaic Effect in CH_3_NH_3_PbI_3_ and CH_3_NH_3_PbI_3-x_Cl_x_. *J. Phys. Chem. Lett.* **6**, 31 (2015)
